# Dual-Modified Lignin-Assembled Multilayer Microsphere with Excellent Pb^2+^ Capture

**DOI:** 10.3390/polym14142824

**Published:** 2022-07-11

**Authors:** Zhaohui Zhang, Yehong Chen, Chaojun Wu

**Affiliations:** 1 Key Laboratory of Pulp and Paper Science & Technology of Ministry of Education, Qilu University of Technology (Shandong Academy of Sciences), Jinan 250353, China; zzh_17@163.com; 2 State Key Laboratory of Biobased Material and Green Papermaking, Qilu University of Technology (Shandong Academy of Sciences), Jinan 250353, China; 3 Faculty of Light Industry, Qilu University of Technology (Shandong Academy of Sciences), Jinan 250353, China

**Keywords:** lignin, adsorption, waste water treatment, heavy metal

## Abstract

With the continuous research on lignin-based sorbents, there are still limitations in the research of spherical sorbents with a high adsorption capacity for Pb^2+^. In order to solve the problem of low adsorption effect, alkali lignin (AL) was modified and assembled to increase the adsorption active sites. In this work, we used dual-modified lignin (DML) as a raw material to assemble a singular lignin-based multilayer microsphere (LMM) with sodium alginate (SA) and dopamine. The prepared adsorbent had various active functional groups and spherical structures; the specific surface area was 2.14 m^2^/g and the average pore size was 8.32 nm. The adsorption process followed the Freundlich isotherm and the second-order kinetic model. Therefore, the LMM adsorbed Pb^2+^ ascribed by the electrostatic attraction and surface complexation; the adsorption capacity was 250 mg/g. The LMM showed a selective adsorption performance for Pb^2+^ and the adsorption capacity followed the order Pb^2+^ (187.4 mg/g) > Cu^2+^(168.0 mg/g) > Mn^2+^(166.5 mg/g). After three cycles, the removal efficiency of Pb^2+^ by the LMM was 69.34%, indicating the reproducibility of LMM.

## 1. Introduction

Lead ion (Pb^2+^) is one of the most common toxic pollutants in industrial wastewater [1]. Due to the non-degradability of Pb^2+^, it can accumulate in the human body through the food chain and accordingly become hazardous for human health. The World Health Organization (WHO) stipulates that the level of lead ions in drinking water should be less than 0.01 mg/L. Moreover, when the daily intake of lead exceeds 0.3 mg, it is considered harmful to human health [2,3]. When the contents of lead ions in the body exceed a certain amount, it makes the human body have kidney failure, high blood pressure, and psychiatric disorders; at the same time, when excess lead ions accumulate in bones, brain, and muscles, they can cause serious developmental disorders, injury, disease, and even death [4]. As a consequence, effectively removing the Pb^2+^ from wastewater has drawn the attention of researchers. In the process of continuous research, coagulation, ion exchange, reverse osmosis, membrane separation, and adsorption are the common wastewater treatment methods [5]. Meanwhile, the adsorption method exhibits broad application prospects, which are due to high efficiency, low cost, recyclability, easy operation, etc. [6].

In previous studies, few researchers used dopamine as a cross-linking agent to assemble adsorbents in the field of lignin-based adsorbent materials. As an important neurotransmitter, dopamine plays an important role in many physiological processes [7], For example, it plays an important role in the modulation of neuroplasticity [8], and Matt et al. found that dopamine also has an effect on immune cell dysfunction in diseases [9]. Landgraf et al. also found a role of dopamine in the circadian rhythm [10]. In terms of chemical structure, it is 3,4-dihydroxy phenylethylamine, which belongs to the catecholamine group as it has a catechol ring and an amine side chain [11]. Such a chemical structure provides the possibility for it to be used as a cross-linking agent.

Sodium alginate (SA) is widely used in the preparation of adsorbents. SA is a biopolymer extracted from brown algae. It has wound-healing properties, good moisture absorption, and high viscosity in water, and it is also a non-toxic, naturally biodegradable green material [12,13]. Researchers found that the SA molecular chain contained many carboxyl and hydroxyl residues, which led to its ability to combine with additional ions to form gels, and the common ion was calcium ion [14]. By cross-linking calcium ions, a microsphere was formed, but the gel spheres had disadvantages, such as large pores, poor strength, and poor stability, so researchers often use blending and chemical modification to improve these properties [13].

As a natural polymer material, lignin not only has a three-dimensional amorphous network structure, but also is the only extendable aromatic polymer [15,16]. According to statistics, 700,000 tons of lignin was annually produced from the pulp and paper industry [17]. Unfortunately, there are still great restrictions on the value-added utilization of lignin [18,19], and more than 95% of lignin is not effectively utilized, resulting in environmental pollution [20]. Alkali lignin (AL), as a by-product of alkali pulping, can cause serious pollution of the water environment and pose a threat to human health if humans cannot properly handle it. Therefore, it is necessary to treat alkali lignin effectively. In addition, AL contains a large number of active functional groups, such as phenolic hydroxyl group and methoxy group, on its surface, so it can remove heavy metal ions in wastewater by chemical reaction. This effectively realizes the value-added utilization of lignin and avoids the waste of biomass energy. However, it was found that the adsorption efficiency of alkali lignin to heavy metal ions was not significant, so it was indispensable to carry out the chemical treatment of AL [21]. The researchers obtained modified lignin-based adsorbents by grafting the copolymerization, oxidation, esterification, sulfonation, and amination of lignin, and prepared lignin-based composite adsorbents by chemical recombination with other materials (such as chitosan, chitin, etc.). Moreover, using lignin, chitin, sodium alginate, and other green polymers to prepare heavy metal adsorbents can not only effectively avoid the pollution caused by these substances to the natural environment, but also purify the polluted wastewater [22]. For example, the maximum adsorption capacity of the lignin-based magnetic adsorbent prepared by Zhou et al., using melamine chloride as a cross-linking agent for Pb^2+^, was 111.23 mg/g [15]. Yan et al. obtained lignin (LGN) and GO (graphene oxide) composite nanospheres using the self-assembly method, and the maximum adsorption capacity was 368.78 mg/g for Cr^6+^ [23]. Popovic et al. prepared an amino-functionalized lignin-based microsphere, which showed a favorable adsorption performance for Cd^2+^ (74.84 mg/g), Cr^6+^ (54.20 mg/g), As^5+^ (53.12 mg/g), and Ni^2+^ (49.42 mg/g) [24]. Zhang et al. prepared functionalized lignin-based hybrid magnetic nanoparticles to adsorb various heavy metal ions; it showed the adsorption capacity of 150.33 mg/g and 70.69 mg/g for Pb^2+^ and Cu^2+^, respectively [25]. The lignosulfonate biosorbent (CLLS) prepared by Zhang et al. also had a favorable adsorption effect on lead ions, with a maximum adsorption capacity of 64.9 mg/g [26]. Chen et al. used MoS_2_ to react with lignin to obtain MoS_2_-lignin-derived carbon nanocomposites, which exhibited an outstanding adsorption performance for Cr^6+^ (198.70 mg/g) [27]. These treatment methods can effectively improve the adsorption effect of heavy metal ions. However, it was found that the lignin-based spherical structure demonstrated excellent adsorption efficiency for Cr^6+^, and there are few studies on the adsorption of lead ions. In recent years, spherical particles have attracted extensive attention because of their regular shape, uniform size, and positive fluidity. Moreover, they also have the advantages of sustainability and easy preparation [28]. According to the uniqueness of the spherical structure, we used a lignin-based spherical adsorbent to study the adsorption of Pb^2+^. In addition, compared with other biomaterials, lignin is abundant in nature, biodegradable, rich in content, environmentally friendly, and biocompatible [29]. As the spherical lignin particles also have the advantage of biodegradability, it has broad application prospects in the field of biological materials [28]. Therefore, on the basis of previous studies, in order to achieve an effective removal of Pb^2+^ in wastewater and improve the adsorption capacity of adsorbent, we tried to combine dual-modified lignin (DML), dopamine, and sodium alginate to obtain a singular lignin-based multilayer microsphere (LMM). In addition, in view of current studies, spherical adsorption materials based on lignin are mainly monolayer and there is still a lack of studies on double-layer microspheres. This study also provides a new idea for the assembly of lignin-based double-layer heavy metal adsorption materials. At the same time, compared with other lignin-based spherical adsorption materials, it also effectively improves the adsorption performance of Pb^2+^.

In the present study, we assembled lignin multilayer microspheres (LMM) using dopamine and sodium alginate for the first time. In order to successful obtained the LMM, we used dual-modified lignin with different carboxyl content values (1.15 and 1.57 mmol/g) to react with dopamine, then assembled with SA. Finally, the LMM not only had multilayer microspheres structures, but had mounts of active groups after modification. Consequently, this work researched the influence of time, pH, temperature, and initial concentration for the adsorption capacity. Meanwhile, we also evaluated the ion selectivity and recyclability of the LMM. This kind of lignin-based adsorbent with high absorbability, multi-absorbability, and simple preparation not only further realized the value-added utilization of lignin, but also demonstrates broad application prospects in wastewater treatment.

## 2. Materials and Methods

### 2.1. Materials

The alkali lignin (AL) in the present study was obtained from the Shanghai Trading, Co., Ltd. (Shanghai, China). and the ClCH_2_COOH, 98%H_2_SO_4_, and HCHO were derived from the Sinopharm Chemical Reagent Co., Ltd. (Shanghai, China). CS_2_, NaOH, sodium alginate, and NaCl were purchased from the Tianjin Damao Chemical Reagent Factory (Tianjin, China). The dopamine hydrochloride was collected from Shanghai Macklin Biochemical Co., Ltd. (Shanghai, China). All reagents were analytically pure.

### 2.2. Methods

**Figure 1:**
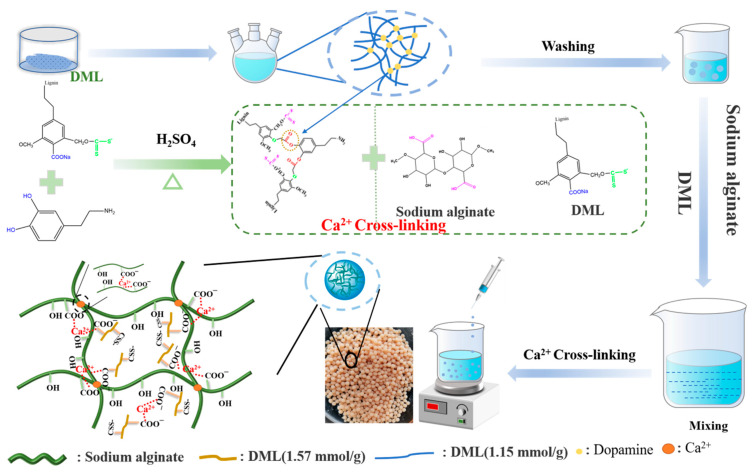
The preparation of the LMM.

The preparation of lignin multilayer microspheres (LMM) is shown in Figure 1 and the preparation of dual-modified lignin (DML) followed previous methods [30]. After that, the different carboxyl contents of DML were obtained by adjusting the ratio of chloroacetic acid and NaOH. 

LMM was prepared using a two-step method including esterification and cross linking: (i) Amounts of 0.1 g DML (carboxyl content = 1.15 mmol/g), 10 mL 10% (*w*/*v*) dopamine hydrochloride, and 20 mL 72% H_2_SO_4_ were poured into a three-necked flask, and the mixture reacted for 5 h at 60 °C. (ii) The lignin prepared above and DML (carboxyl content = 1.57 mmol/g) was combined with sodium alginate in a beaker and was stirred for 3 h until the solution was fully mixed. Then, using an injector, this solution was added the 2% (*w*/*v*) Ca^2+^ solution and left for 12 h. Finally, these spheres were freeze-dried to obtain the LMM.

### 2.3. Characterization of the LMM

Fourier transform infrared (FT-IR) spectroscopy was used to analysis the structure of the LMM (ALPHA Infrared spectrometer Shimazu Corporation, Japan) and the microstructure of the AL and LMM were recorded on an SEM (HitachiRegulus8220 Hitachi, Japan). The S_BET_ data was obtained using a Mike ASAP 3020 analyzer (McMurtic Instruments Co., LTD., USA). The XRD data was received using a multifunctional powder XRD instrument (D8-ADVANCE, Bruker, Germany) and the chemical structure variation of the LMM before and after adsorption was tested using an XPS instrument (ESCALABXi+ American Brooker Company, USA). A Shanghai Ray Magnetic Multi-Parameter Analyzer (DZS-706) exhibited the carboxyl content of the LMM. The Pb^2+^ concentrations were acquired using a GGX-600 AAS flame atomic absorption spectrometer (Beijing Haiguang Instrument Co., LTD., Beijing, China).

### 2.4. Adsorption Experiments

In order to research the effect of dosage, pH, temperature, duration, and concentration for the adsorption performance, the 30 mg/L Pb^2+^ solution was poured into a 150 mL conical flask with different conditions to study the adsorption capacity. The residual Pb^2+^ concentration was recorded using the atomic adsorption method. The adsorption capacity (q_e_) of the LMM followed the expression:

(1) $$q_{e}=\frac{\left(C_{0}-C_{e}\right)V}{M}$$

Meanwhile, the removal efficiency (E) of the LMM followed the expression:

(2) $$E=\frac{\left(C_{0}-C_{e}\right)}{C_{0}}\times 100\%$$

where C_0_ (mg/L) and C_e_ (mg/L) represent the Pb^2+^ solution concentration before and after adsorption, respectively; V (L) is the volume of solution; M (g) is the mass of the adsorbent. The experiments were repeated three times.

### 2.5. The Reproducibility of the LMM

The recoverability of the LMM was measured by adsorption-desorption experiments. In this process, 100 mg LMM was added to the 50 mL Pb^2+^ solution (30 mg/L), then left to react for 4 h at pH 5. After finishing adsorption experiments, the lead-loaded LMM was obtained by filtration, soaking it in 50 mL 1M HCl for 60 min, and repeating twice. After that, the sample was washed in deionized water, underwent centrifugation, and was dried at 80 °C for 12 h. Finally, this adsorbent adsorbed Pb^2+^ again.

## 3. Results and Discussion

### 3.1. The Determination of Carboxyl Content in DML

**Figure 2:**
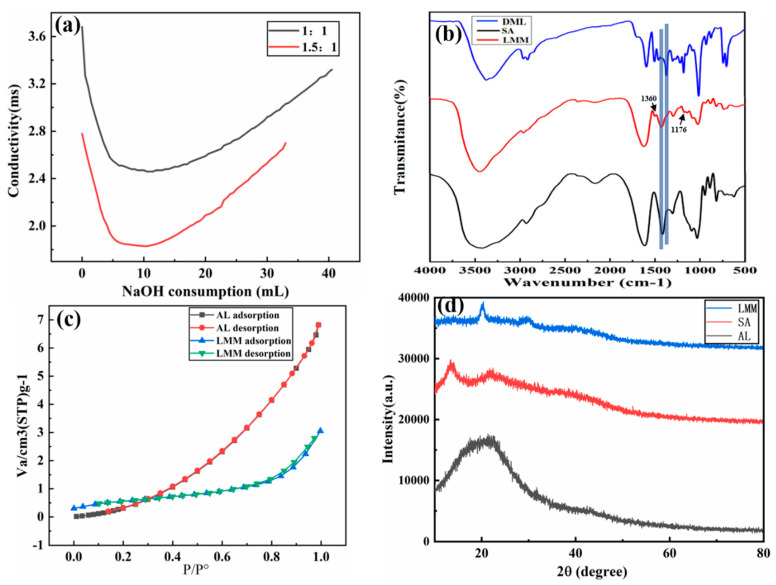
(**a**) Carboxyl content of lignin-based multilayer microspheres (LMM); (**b**) FT-IR spectrogram of alkali lignin (AL), sodium alginate (SA), and LMM; (**c**) N_2_ adsorption-desorption isotherms of LMM; (**d**) XRD spectra of AL, SA, and LMM.

In this work, we used conductivity titration to measure the carboxyl content of the DML. When the ratio of NaOH and chloroacetic acid changed, the content of carboxyl groups in the DML also changed. When the conditions of NaOH/chloroacetic acid content ratio were 1:1 and 1.5:1, the NaOH consumption-conductivity curve was constructed (as shown in Figure 2a), and the carboxyl content of different DMLs was calculated using a carboxyl content calculation formula (Equation (3)). It was found that the carboxyl content of the DML were 1.57 and 1.15 mmol/g.

The carboxyl content calculation formula is as follows:

(3) $$\mathrm{Carboxyl}\;\mathrm{content}=\frac{(0.04\;\mathrm{mol}/L\times \left(V_{B}-V_{A}\right)\;\mathrm{mL}}{O.2\;g}$$

where V_B_ is the consumption of NaOH at the end of conductivity equilibrium and V_A_ is the consumption at the beginning of conductivity stabilization.

### 3.2. The Characterization of the LMM

As shown in Figure 2b, the spectra of the DML, SA, and the LMM are introduced. As can be seen from the spectra, the structure of adsorption changed significantly and the peak of sodium alginate at 1407 cm^−1^ shifted towards 1429 cm^−1^ due to the symmetric deformation of -COO-, indicating the cross-linking of carboxylic acid base group in the system [31]. At the same time, the changes at 1282, 1220, 1087, and 1031 cm^−1^ also explain the successful cross linking between sodium alginate and DML. In addition, the peak at 1176 cm^−1^ indicates C-O-C stretching vibration in the ester group, and the weak peak at 1360 cm^−1^ indicates the presence of C-N in dopamine, which also indicates the chemical reaction between dopamine and DML.

Furthermore, Figure 2c displays the N_2_ adsorption-desorption curve, which shows that the specific surface area of the LMM was 2.14 m^2^/g. We found that the specific surface area of the LMM was higher than the AL (1.66 m^2^/g), while the average pore size of the LMM (8.32 nm) was lower than the AL (10.5 nm). These parameters indicate that the microstructure and average pore size of specific surface area of AL were changed by modification.

Similarly, the XRD data showed the crystal structure of the AL, sodium alginate, and the LMM; as can be seen from Figure 2d, with the addition of sodium alginate, the peak value of LMM was weakened but still maintained non-crystal structure, showing that the crystal structure of LMM did not change significantly after modification and curing, and that it had a non-crystal structure [32].

**Figure 3:**
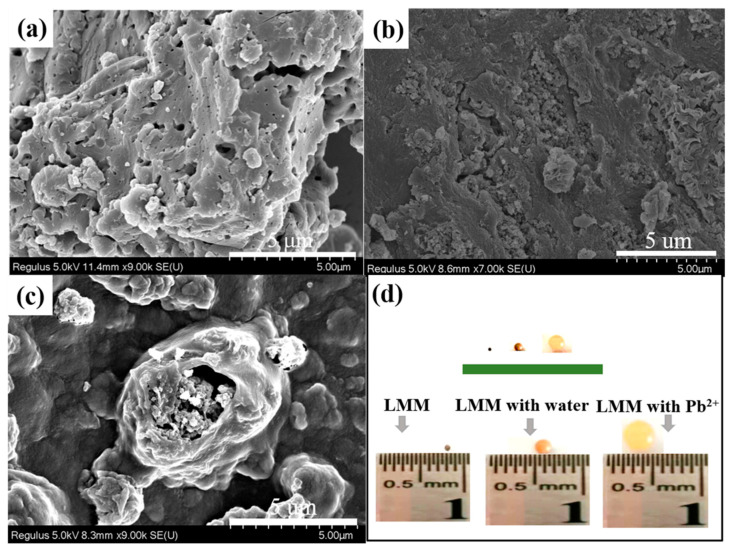
SEM images of (**a**) alkali lignin (AL); (**b**) lignin-based multilayer microspheres (LMM); (**c**) adsorbing Pb^2+^; (**d**) size of LMM.

In order to research the microscopic surface morphology of the AL and LMM, this work used SEM to discuss the surface structure. It is shown in Figure 3 that the micromorphology evidently changed and the LMM had a spheroidal structure, which differed from the blocky structure of the AL. From Figure 3b, it can be seen that the LMM did not have rough morphology; however, pore structures existed on the surface of the LMM and it also had aggregation of microspheres in the ores. This shows that the multilayer microspheres were successfully prepared using sodium alginate and dopamine. Figure 3c shows the microscopic surface morphology of Pb^2+^ adsorbed by LMM. In addition, Figure 3d displays the diameter changes of the LMM, the LMM with deionized water, and the LMM with lead ions. These results show the successful adsorption of Pb^2+^ by the LMM.

### 3.3. Adsorption Efficiency of the LMM for Pb^2+^

**Figure 4:**
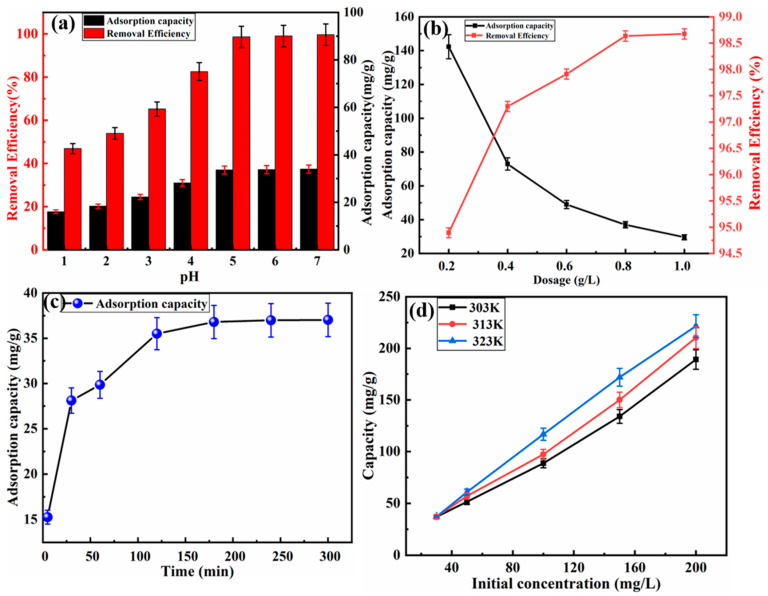
Influence of (**a**) solution pH, (**b**) adsorbent dosage, (**c**) time, and (**d**) temperature on adsorption capacity of lignin-based multilayer microspheres.

To discuss the influence of pH on the adsorption capacity of the LMM, the adsorption experiments was carried out with different pH values, and the mass of the LMM was 0.8 g/L at 30 °C for 4 h. It can be seen from Figure 4a that the LMM had an adsorption effect at pH 2, and with the increase of the pH values, the removal efficiency and capacity evidently increased. In addition, when the pH was 5, the removal efficiency and capacity were 98.66% and 36.99 mg/g, respectively. When the pH was higher than 6, the removal efficiency and capacity reached 99.67% and 37.38 mg/g, respectively. These results were caused by the production of Pb(OH)_2_ [33]. In conclusion, this adsorption test allowed the selection of pH 5 as the best condition in next step.

The dosage of the LMM was also an important role in adsorption capacity. Therefore, the test with varying dosages (0.2, 0.4, 0.6, 0.8, and 1.0 g/L) was conducted to research the adsorption capacity for Pb^2+^. As it can be seen from Figure 4b, the increased dosage of the LMM would decrease the adsorption capacity for Pb^2+^, while the adsorption efficiency would increase, and the maximum adsorption efficiency and capacity were 99% and 142.3 mg/g, respectively. Therefore, 0.8 g/L LMM was selected as the optimal condition in the following experiments.

Figure 4c exhibits the effect of time on the adsorption capacity. The adsorption capacity of the LMM rose quickly when the adsorption time ranged from 0 to 30 min, and then the adsorption capacity increased slowly until 180 min. This phenomenon may be caused by the large number of active sites on the surface of the LMM. Meanwhile, the adsorption process reached sorption equilibrium as the adsorption time reached 180 min and the adsorption capacity was 37.1 mg/g.

The temperature was a crucial effect for the adsorption performance. Figure 4d showed the temperature on the removal of Pb^2+^ by the LMM. The adsorption capacity increased when the temperature increased, indicating that the adsorption process was an endothermic process; high temperature was more conducive to adsorption.

### 3.4. Adsorption Kinetics

In order to explore the adsorption mechanism of the LMM adsorbing Pb^2+^, the adsorption kinetics were analyzed by determining the adsorption capacity and time ranging from 30 to 300 min at pH 5 and 30 °C. Then, the experiment data were fitted with the pseudo-first-order, pseudo-second-order, and intraparticle diffusion kinetics models [34]. 

The adsorption capacity of the LMM at time t was obtained by this equation:

(4) $$q_{t}=\frac{\left(C_{0}-C_{t}\right)\times V}{M}$$

where C_0_ is the initial concentration of Pb^2+^ (mg/L), Ct is the concentration of Pb^2+^ at time t (mg/L), V is the volume of the solution (L), and the M is the mass of the LMM (g).

The pseudo-first-order is expressed using the following equation:

(5) $$\log\left(q_{e}-q_{t}\right)={\mathrm{logq}}_{e}-\frac{k_{1}}{2.303}t$$

The pseudo-second-order is calculated as follows:

(6) $$\frac{t}{q_{t}}=\frac{1}{k_{2}q_{e}^{2}}+\frac{1}{q_{e}}t$$

Meanwhile, the intraparticle diffusion model is expressed by the equation [35]:

(7) qt=kpt12+C

where q_t_ and q_e_ (mg/g) are the adsorption capacities at time t and equilibrium, respectively; k_1_ (1/min) and k_2_ (g/mg min) are the pseudo-first-order and pseudo-second-order model rate constants, respectively; k_p_ is the intraparticle diffusion rate constant [mg/(g min^1/2^)]; and C represents the thickness of the boundary layer.

**Figure 5:**
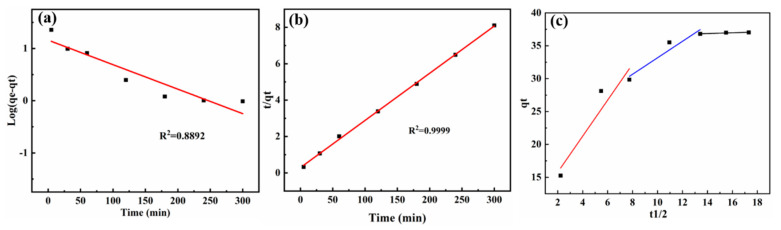
Fitting adsorption kinetics of (**a**) pseudo-first-order model, (**b**) pseudo-second-order model, and (**c**) intraparticle diffusion model.

The relation parameters of the pseudo-first-order, pseudo-second-order, and the intraparticle diffusion model are listed in Table 1. The fitting curves (Figure 5) exhibit that the adsorption process followed pseudo-second-order, and the R^2^ of the pseudo-first-order and pseudo-second-order were 0.9696 and 0.9992, respectively. In addition, the R^2^ of the intraparticle diffusion model were 0.9053, 0.9274, and 0.8944, and the C was different from zero. According these results, it could be concluded that the adsorption process of the LMM for Pb^2+^ was mainly chemical adsorption and the interaction between the LMM and Pb^2+^ was not a simple diffusion [36].

### 3.5. Adsorption Isotherm and Thermodynamics

The relation between LMM and Pb^2+^ was investigated using two equilibrium isotherm models with the Langmuir model and Freundlich model [37].

The Langmuir model can be expressed as follows:

(8) $$\frac{C_{e}}{q_{e}}=\frac{1}{K_{L}\cdot q_{m}}+\frac{C_{e}}{q_{m}}$$

where C_e_ is the concentration of Pb^2+^ at equilibrium (mg/L), q_e_ is the equilibrium adsorption capacity (mg/g), and K_L_ (L/mg) (L/g) is the constant of the Langmuir model.

The Freundlich model is obtained as the equation:

(9) $${\mathrm{lnq}}_{e}={\mathrm{lnK}}_{F}+\frac{1}{n}\cdot {\mathrm{lnC}}_{e}$$

where K_F_ [(mg/g) (L/mg)^1/n^] and n are the Freundlich constants.

**Figure 6:**
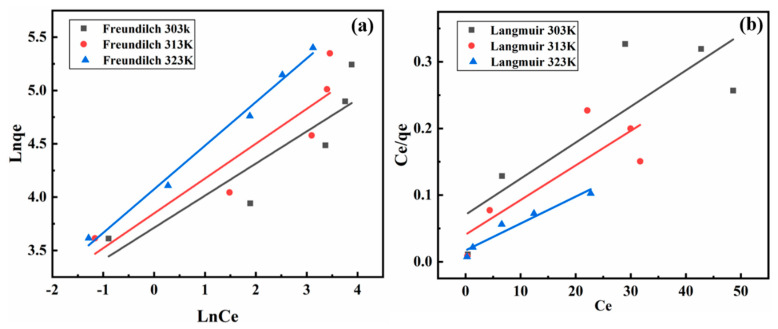
Fitting adsorption equilibrium isotherms of (**a**) Freundlich model and (**b**) Langmuir model.

The adsorption isotherm curves of the LMM for Pb^2+^ is demonstrated in Figure 6 and the parameters are exhibited in Table 2. It can be seen that the R^2^ of Freundlich model was higher than the Langmuir model. Therefore, the multi-layer chemical adsorption could be used to display the adsorption process of Pb^2+^ by the LMM. According to the Langmuir model parameters, it could be obtained that the maximum adsorption capacity was 250 mg/g. In addition, because of the 1/n was between 0 and 1, it illustrated that the Pb^2+^ could easily react with the LMM [38].

Furthermore, this material could be comparable to the other adsorbent materials reported in the past. Table 3 shows the adsorption capacity of the different adsorbent materials that adsorbed Pb^2+^. It can be seen that the adsorption capacity of LMM for Pb^2+^ was at a medium level, and the adsorption performance was better than that of other spherical adsorbents. This may be because the double spherical structure provides more adsorption sites for lead ion adsorption. Moreover, the LMM has the advantage of low cost, easy preparation, and high hydrophobicity, which makes the LMM a wide application prospect.

### 3.6. Thermodynamics of the LMM Adsorption

In order to investigate the thermodynamics of the LMM adsorption for Pb^2+^, the three thermodynamics parameters, including ΔG (kJ/mol), ΔH (kJ/mol), and ΔS (kJ/mol/K), were obtained using the following equations [43]:

(10) $$\mathrm{Ln}\left(K_{d}\right)=-\frac{\Delta H}{\mathrm{RT}}+\frac{\Delta S}{R}$$

(11) ΔG=ΔH−TΔS

where K_d_ (mL/g) is the equilibrium constant, T (K) is the temperature, and R is the gas constant (J/mol/K).

Table 4 exhibits the thermodynamics parameters about the adsorption of the LMM for Pb^2+^. ΔG < 0 showed that the adsorption process was a spontaneous reaction and the rates of ΔG were −28.89, −30.38, and −31.87 KJ/mol at 303, 313, and 323 K, respectively. These results indicate that the adsorption process spontaneously enhanced when the temperature increased.

In addition, the positive value of ΔH explained that the adsorption process was an endothermic process and this result describes the reason that the adsorption capacity increased as the temperature raised. Similarly, the positive ΔS suggested the adsorption reaction led to the disordered movement of molecules and it was an entropy-driven process. Meanwhile, the experiment results of the effect of temperature with adsorbing Pb^2+^ by the LMM fitted well with this analysis.

### 3.7. Recyclability of the LMM

**Figure 7:**
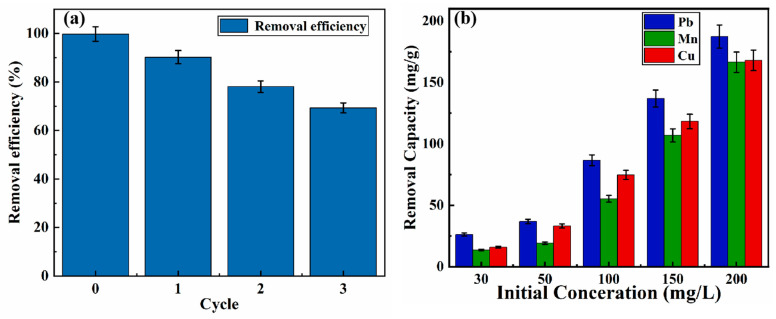
(**a**) Adsorption and desorption cycles of lignin-based multilayer microspheres (LMM) for Pb^2+^ and (**b**) selectivity of LMM for Pb^2+^, Cu^2+^, and Mn^2+^.

The recyclability of Pb^2+^ using the LMM was analyzed using an adsorption-desorption experiment. As shown in Figure 7a, the adsorption efficiency of the LMM for Pb^2+^ decreased slowly with cycles increased. It can be seen that the removal efficiency was 98.82% in the initial adsorption; after the three cycles, the removal efficiency decreased to 69.34%. These results show that the LMM could be reused to adsorb Pb^2+^. Although the adsorption efficiency certainly declined, the LMM showed regeneration properties. At the same time, we analyzed the reasons for the decrease of LMM adsorption performance, which may be caused by the destruction of LMM’s chemical structure and the incomplete desorption of Pb^2+^. Therefore, in the follow-up study, we will conduct experiments and solve problems based on these reasons.

### 3.8. Selectivity of the LMM Adsorption

The selectivity of the LMM was accessed using the adsorption capacity of other heavy metal ions by the LMM. The adsorption experiment result is shown in Figure 7b; it indicates that the adsorption capacity of the LMM for Pb^2+^, Cu^2+^, and Mn^2+^ follow the order Pb^2+^ > Cu^2+^ > Mn^2+^. It was caused by the differences in their corresponding electronegativities (Pb^2+^ = 2.33, Cu^2+^ = 2.0, and Mn^2+^ = 1.55) [44]. Moreover, the appearance of this phenomenon could also be explained by the Pearson’s hard/soft acid/base (HSAB) theory. According to acid base classification, Pb^2+^ was considered to a softer acid than Cu^2+^ and Mn^2+^, and COO-, CSS¯, C/N, and NH_2_ contained in LMM can be used as soft bases, thus producing a better affinity for lead ions [15,45]. Meanwhile, it was also found that the LMM exhibited good adsorption capacity for three heavy metal ions and the maximum adsorption capacity of the LMM for Pb^2+^, Cu^2+^, and Mn^2+^ were 187.4, 168.0, and 166.5 mg/g, respectively. Thus, it could be concluded that the LMM had versatility and the adsorption performance of Pb^2+^ was the best among the three heavy metal ions.

### 3.9. Adsorption Mechanism

**Figure 8:**
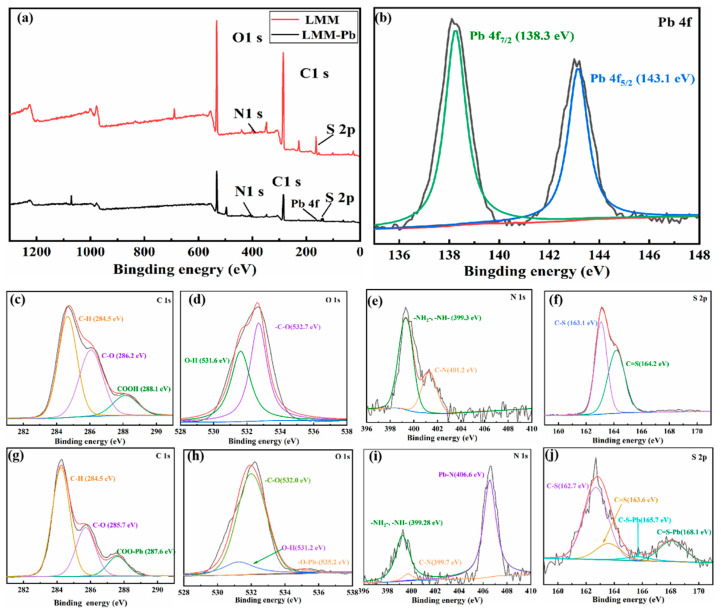
(**a**) XPS spectra of lignin-based multilayer microspheres (LMM) and LMM-Pb; (**b**) characteristic peaks of Pb and fitting peaks of (**c**,**g**) C 1s, (**d**,**h**) O 1s, (**e**,**i**) N 1s, and (**f**,**j**) S 2p.

The adsorption mechanism of the LMM for Pb^2+^ could be explored by XPS analysis. Figure 8 suggests the changes of the LMM and the LMM involving Pb^2+^. The peaks of N, S, and Pb explain the successful preparation of the LMM and the Pb^2+^ adsorption. The 4f^5/2^ and 4f^7/2^ signals appearing in Figure 8b illustrate the adsorption of the LMM for Pb^2+^. Meanwhile, the peaks of the C-H and C-O shifted from 284.5 eV and 286.2 eV to 284.5 eV and 285.4 eV, respectively, after adsorption Pb^2+^, and the peaks of COOH shifted from 288.1 eV to 287.6 eV after adsorption Pb^2+^, which would be caused by the appearance of COO-Pb (Figure 8c,g) [30]. Additionally, the -C-O and O-H peaks were 531.6 eV and 532.7 eV, respectively; after adsorbing Pb^2+^, the -C-O and O-H peaks were 531.2 eV and 532.0 eV, respectively. Moreover, the 535.2 eV signal explains the existence of -O-Pb [46]. The peaks of the N 1s spectra at 399.3 eV and 401.2 eV were caused by the -NH_2_-, -NH-, and C-N bonds of the LMM. Then, the peaks of -NH_2_-, -NH-, and C-N changed to 399.28 eV and 399.7 eV and the new peaks appeared at 406.6 eV (Pb-N); these results demonstrate that the nitrogenous functional groups participated in the adsorption process of the LMM for Pb^2+^ [2,15,18]. In addition, the S 2p XPS spectra could be fitted into two peaks at 163.1 eV and 164.2 eV, which were C-S and C=S bonds. After the LMM reacted with Pb^2+^, the peaks of C-S and C=S were 162.7 eV and 163.6 eV, respectively. The S 2p spectra produced two new peaks at 165.7 eV and 168.1 eV, which were appointed to C-S-Pb and C=S-Pb [47], respectively. In conclusion, the emergence of new peaks of Pb and chemical bonds reacting with Pb can be seen from Figure 8. Meanwhile, the other chemical bonds had significant changes after adsorbing lead ions. These consequences illustrated the formation of coordination complexes between Pb^2+^ and the LMM.

**Figure 9:**
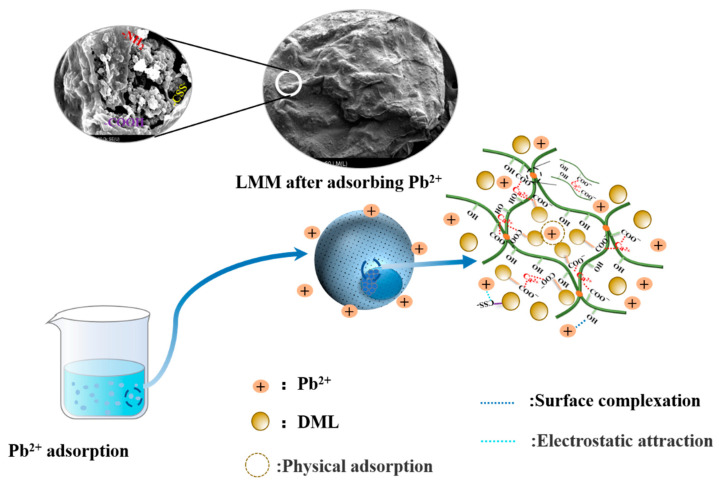
Adsorption mechanism of lignin-based multilayer microspheres (LMM) for Pb^2+^.

In conclusion, according to the analysis of the XPS spectra, the main adsorption process of the LMM for Pb^2+^ was chemical adsorption, including complexation and electrostatic attraction. On the other hand, the SEM images and specific surface area parameters also showed that the adsorption process involved physical adsorption. Meanwhile, the pore size of LMM was 8.32 nm while the diameter of the lead ion was about 0.15 nm. This indicated that Pb^2+^ could better enter the interior of LMM, which was more conducive to the physical adsorption and internal chemical reaction of Pb^2+^ on the LMM. Therefore, the LMM adsorbed the Pb^2+^ mechanism mainly using chemical adsorption because of the existence of active groups and there was some physical adsorption in the adsorption process because of the microstructure (as shown in Figure 9).

## 4. Conclusions and Future Perspectives

Lignin-based bilayer microspheres prepared by the green method have the advantages of easy preparation, renewability, high adsorption, and versatility. In the present study, dual-modified lignin was assembled by dopamine and sodium alginate to obtain a double-layer lignin microsphere. The diameter of the microsphere obtained was about 800 µm, the specific surface area was 2.14 m^2^/g, and the average pore size was 8.2 nm. The adsorption process followed the pseudo-second-order kinetic equation and Freundlich model, indicating that the adsorption was multi-layer adsorption dominated by chemical adsorption. The maximum adsorption capacity of Pb^2+^ by LMM was 250.0 mg/g, determined by Langmuir model analysis and the adsorption of Pb^2+^ by LMM was mainly composed of electrostatic attraction and surface complexation. In addition, it was found that after three cycles, the adsorption performance of LMM for Pb^2+^ decreased from 98.82% to 69.34%, indicating that LMM also had a certain reproducibility. The LMM showed high adsorption performance for various heavy metal ions, including Pb^2+^, Cu^2+^, and Mn^2+^, and the adsorption capacity (at 303 K, initial concentration = 30 mg/L, pH = 5, time = 180 min) followed the order of Pb^2+^(187.4 mg/g) > Cu^2+^(168.0 mg/g) > Mn^2+^(166.5 mg/g). The lignin bilayer microspheres prepared from low-cost and readily available lignin have great potential in the removal of heavy metal ions. In the next research process, the assembly and adsorption properties of lignin-based adsorption materials will be further studied to expand their application in water treatment materials. At the same time, the multi-functional use of lignin-based adsorbent materials further realizes the value-added utilization of lignin.

## Figures and Tables

**Table 1:** The adsorption kinetics parameters of the LMM for Pb^2+^.

Model	Pseudo-First-Order		
C_0_ (mg/L)	q_e_ (mg/g)	k_1_ (min^−1^)	R^2^
30	22.08	0.02349	0.9696
	Pseudo-second-order		
C_0_ (mg/L)	q_e_ (mg/g)	k_2_ (g/mg min)	R^2^
30	38.91	0.001929	0.9999
	intraparticle diffusion model		
	C	K_p_	R^2^
Step 1	10.29	2.739	0.9053
Step 2	20.65	1.252	0.9274
Step 3	36.04	0.05840	0.8944

**Table 2:** The equilibrium isotherm models parameters for Pb^2+^ by the LMM.

Model	Langmuir Model		
T (K)	q_max_ (mg/g)	K_L_ (L/mg)	R^2^
303	185.19	0.07649	0.7248
313	204.81	0.1012	0.7721
323	250.0	0.2353	0.9422
	Freundlich model		
T (K)	K_F_ (L/g)	1/n	R^2^
303	40.85	0.3011	0.8130
313	46.53	0.3266	0.8783
323	58.56	0.4088	0.9900

**Table 3:** The comparisons of the adsorption capacity of Pb^2+^ by other adsorbents.

Adsorbent	Adsorption Capacity (mg/g)	Reference
chitosan/lignosulfonate hydrogel	525	[18]
Bio-sourced phenolic foams	100.9	[39]
Cross-linked lignosulfonate bio-adsorbent	64.9	[26]
Modified alkaline lignin	73.7	[40]
Functionalized lignin-based adsorbent	91.4	[41]
Lignin-based hybrid magnetic nanoparticles	150.33	[25]
Magnetic polyethyleneimine lignin	96.60	[42]
LMM	250.0	This work

**Table 4:** The Thermodynamics paraments of Pb^2+^ adsorption by the LMM.

T (K)	ΔG (KJ/mol)	ΔH (KJ/mol)	ΔS (KJ/mol/K)
303	−28.89	16.26	0.149
313	−30.38		
323	−31.87		

## Data Availability

Data is contained within the article.

## References

[1] Yaqoob A.A., Parveen T., Umar K., Mohamad Ibrahim M.N. (2020). Role of Nanomaterials in the Treatment of Wastewater: A Review. Water.

[2] Ji Z., Sun H., Zhu Y., Zhang D., Wang L., Dai F., Zhao Y., Chen L. (2021). Enhanced selective removal of lead ions using a functionalized PAMAM@UiO-66-NH2 nanocomposite: Experiment and mechanism. Microporous Mesoporous Mater..

[3] Kucherova A.E., Romantsova I.V., Burakov A.E., Memetov N.R., Krasnyansky M.N. (2017). Graphene-Based Nanocomposites for Enhanced Pb^2+^ Adsorption. Nano Hybrids Compos..

[4] Yaqoob A.A., Ahmad H., Parveen T., Ahmad A., Oves M., Ismail I.M.I., Qari H.A., Umar K., Mohamad Ibrahim M.N. (2020). Recent Advances in Metal Decorated Nanomaterials and Their Various Biological Applications: A Review. Front. Chem..

[5] Zhou H., Shi X., Wu W., An X., Tian Y., Qiao Y. (2020). Facile preparation of lignosulfonate/N-methylaniline composite and its application in efficient removal of Cr(VI) from aqueous solutions. Int. J. Biol. Macromol..

[6] Aaa B., Shms B., Aay C., Mnmi C. (2021). Synthesis and characterization of GO-Ag nanocomposite for removal of malachite dye from aqueous solution. Mater. Today Proc..

[7] Zhai C., Sun F., Zhang P., Ma H., Song A., Hao J. (2016). Interactions of dopamine and dopamine hydrochloride with ethanol. J. Mol. Liq..

[8] Chen P.S., Asif J., Liu L.C., Wei S.Y., Huai-Hsuan T., Nitsche M.A., Min-Fang K. (2020). Nonlinear Effects of Dopamine D1 Receptor Activation on Visuomotor Coordination Task Performance. Cereb. Cortex.

[9] Matt S.M., Gaskill P.J. (2019). Where Is Dopamine and how do Immune Cells See it? Dopamine-Mediated Immune Cell Function in Health and Disease. J. Neuroimmune Pharmacol..

[10] Landgraf D., Joiner W.J., Mccarthy M.J., Kiessling S., Barandas R., Young J.W., Cermakian N., Welsh D.K. (2016). The mood stabilizer valproic acid opposes the effects of dopamine on circadian rhythms. Neuropharmacology.

[11] Figueiredo M.L.B., Martin C.S., Furini L.N., Rubira R.J.G., Batagin-Neto A., Alessio P., Constantino C.J.L. (2020). Surface-enhanced Raman scattering for dopamine in Ag colloid: Adsorption mechanism and detection in the presence of interfering species. Appl. Surf. Sci..

[12] Lan W., He L., Liu Y. (2018). Preparation and Properties of Sodium Carboxymethyl Cellulose/Sodium Alginate/Chitosan Composite Film. Coatings.

[13] Hu Y., Chen T., Dong X., Mei Z. (2015). Preparation and characterization of composite hydrogel beads based on sodium alginate. Polym. Bull..

[14] Wang X., Jiang Z., Shi J., Zhang C., Zhang W., Wu H. (2013). Dopamine-Modified Alginate Beads Reinforced by Cross-Linking via Titanium Coordination or Self-Polymerization and Its Application in Enzyme Immobilization. Ind. Eng. Chem. Res..

[15] Zhou X., Jin C., Liu G., Wu G., Huo S., Kong Z. (2021). Functionalized lignin-based magnetic adsorbents with tunable structure for the efficient and selective removal of Pb(II) from aqueous solution. Chem. Eng. J..

[16] Ku A., Aay A., Mnmi A., Tpb C., Tus A. (2021). Environmental applications of smart polymer composites. Smart Polym. Nanocompos..

[17] Popovic A.L., Rusmirovic J.D., Velickovic Z., Kovacevic T., Jovanovic A., Cvijetic I., Marinkovic A.D. (2021). Kinetics and column adsorption study of diclofenac and heavy-metal ions removal by amino-functionalized lignin microspheres. J. Ind. Eng. Chem..

[18] Zhang F., Wang B., Jie P., Zhu J., Cheng F. (2021). Preparation of chitosan/lignosulfonate for effectively removing Pb(II) in water. Polymer.

[19] Pang Y., Chen Z., Zhao R., Yi C., Qiu X., Qian Y., Lou H. (2021). Facile synthesis of easily separated and reusable silver nanoparticles/aminated alkaline lignin composite and its catalytic ability. J. Colloid Interface Sci..

[20] Ge Y., Qin L., Li Z. (2016). Lignin microspheres: An effective and recyclable natural polymer-based adsorbent for lead ion removal. Mater. Des..

[21] Wu F., Chen L., Hu P., Wang Y., Deng J., Mi B. (2021). Industrial alkali lignin-derived biochar as highly efficient and low-cost adsorption material for Pb(II) from aquatic environment. Bioresour. Technol..

[22] Wysokowski M., Klapiszewski L., Moszynski D., Bartczak P., Szatkowski T., Majchrzak I., Siwinska-Stefanska K., Bazhenov V.V., Jesionowski T. (2014). Modification of chitin with kraft lignin and development of new biosorbents for removal of cadmium(II) and nickel(II) ions. Mar. Drugs.

[23] Yan Z., Wu T., Fang G., Ran M., Shen K., Liao G. (2021). Self-assembly preparation of lignin–graphene oxide composite nanospheres for highly efficient Cr(vi) removal. RSC Adv..

[24] Popovic A.L., Rusmirovic J.D., Velickovic Z., Radovanovic Z., Ristic M., Pavlovic V.P., Marinkovic A.D. (2020). Novel amino-functionalized lignin microspheres: High performance biosorbent with enhanced capacity for heavy metal ion removal. Int. J. Biol. Macromol..

[25] Zhang Y., Ni S., Wang X., Zhang W., Lagerquist L., Qin M., Willför S., Xu C., Fatehi P. (2019). Ultrafast adsorption of heavy metal ions onto functionalized lignin-based hybrid magnetic nanoparticles. Chem. Eng. J..

[26] Zhang X., Lu A., Li D., Shi L., Luo Z., Peng C. (2020). Simultaneous removal of methylene blue and Pb(2+) from aqueous solution by adsorption on facile modified lignosulfonate. Environ. Technol..

[27] Chen H., Zhang Z., Zhong X., Zhuo Z., Tian S., Fu S., Chen Y., Liu Y. (2021). Constructing MoS2/Lignin-derived carbon nanocomposites for highly efficient removal of Cr(VI) from aqueous environment. J. Hazard. Mater..

[28] Stanisz M., Klapiszewski L., Kolodynska D., Jesionowski T. (2021). Development of functional lignin-based spherical particles for the removal of vanadium(V) from an aqueous system. Int. J. Biol. Macromol..

[29] Meng Y., Li C., Liu X., Lu J., Cheng Y., Xiao L.P., Wang H. (2019). Preparation of magnetic hydrogel microspheres of lignin derivate for application in water. Sci. Total Environ..

[30] Zhang Z., Wu C., Ding Q., Yu D., Li R. (2021). Novel dual modified alkali lignin based adsorbent for the removal of Pb^2+^ in water. Ind. Crops Prod..

[31] Tao E., Ma D., Yang S., Hao X. (2020). Graphene oxide-montmorillonite/sodium alginate aerogel beads for selective adsorption of methylene blue in wastewater. J. Alloys Compd..

[32] Shi X., Hong J., Li J., Kong S., Song G., Naik N., Guo Z. (2021). Excellent selectivity and high capacity of As (V) removal by a novel lignin-based adsorbent doped with N element and modified with Ca(2+). Int. J. Biol. Macromol..

[33] Liu C., Li Y., Hou Y. (2019). Preparation of a Novel Lignin Nanosphere Adsorbent for Enhancing Adsorption of Lead. Molecules.

[34] Jin C., Zhang X., Xin J., Liu G., Wu G., Kong Z., Zhang J. (2017). Clickable Synthesis of 1,2,4-Triazole Modified Lignin-Based Adsorbent for the Selective Removal of Cd(II). Sustain. Chem. Eng..

[35] Sohni S., Hashim R., Nidaullah H., Lamaming J., Sulaiman O. (2019). Chitosan/nano-lignin based composite as a new sorbent for enhanced removal of dye pollution from aqueous solutions. Int. J. Biol. Macromol..

[36] Wang X., Li X., Peng L., Han S., Hao C., Jiang C., Wang H., Fan X. (2021). Effective removal of heavy metals from water using porous lignin-based adsorbents. Chemosphere.

[37] Qin L., Ge Y., Deng B., Li Z. (2017). Poly (ethylene imine) anchored lignin composite for heavy metals capturing in water. J. Taiwan Inst. Chem. Eng..

[38] Albadarin A.B., Collins M.N., Naushad M., Shirazian S., Walker G., Mangwandi C. (2017). Activated lignin-chitosan extruded blends for efficient adsorption of methylene blue. Chem. Eng. J..

[39] Issaoui H., Sallem F., Lafaille J., Grassl B., Charrier–El Bouhtoury F. (2021). Biosorption of Heavy Metals from Water onto Phenolic Foams Based on Tannins and Lignin Alkaline Liquor. Int. J. Environ. Res..

[40] Wang Q., Zheng C., Zhang J., He F., Yao Y., Zhang T.C., He C. (2020). Insights into the adsorption of Pb(II) over trimercapto-s-triazine trisodium salt-modified lignin in a wide pH range. Chem. Eng. J. Adv..

[41] Jin C., Liu G., Wu G., Huo S., Liu Z., Kong Z. (2020). Facile fabrication of crown ether functionalized lignin-based biosorbent for the selective removal of Pb(II). Ind. Crops Prod..

[42] Zhang X., Li Y., Hou Y. (2019). Preparation of magnetic polyethylenimine lignin and its adsorption of Pb(II). Int. J. Biol. Macromol..

[43] Wang X., Wang Y., He S., Hou H., Hao C. (2018). Ultrasonic-assisted synthesis of superabsorbent hydrogels based on sodium lignosulfonate and their adsorption properties for Ni(2+). Ultrason. Sonochem..

[44] Ge Y., Xiao D., Li Z., Cui X. (2014). Dithiocarbamate functionalized lignin for efficient removal of metallic ions and the usage of the metal-loaded bio-sorbents as potential free radical scavengers. J. Mater. Chem. A.

[45] Bo S., Luo J., An Q., Xiao Z., Wang H., Cai W., Zhai S., Li Z. (2020). Efficiently selective adsorption of Pb(Ⅱ) with functionalized alginate-based adsorbent in batch/column systems: Mechanism and application simulation. J. Clean. Prod..

[46] Zhang D., Wang L., Zeng H., Rhimi B., Wang C. (2020). Novel polyethyleneimine functionalized chitosan–lignin composite sponge with nanowall-network structures for fast and efficient removal of Hg(ii) ions from aqueous solution. Environ. Sci. Nano.

[47] Wang Q., Zheng C., Cui W., He F., Zhang J., Zhang T.C., He C. (2020). Adsorption of Pb^2+^ and Cu^2+^ ions on the CS2-modified alkaline lignin. Chem. Eng. J..

